# Optimizing surgical approaches for anterior communicating artery aneurysms: Development and internal validation of a novel surgical scoring system

**DOI:** 10.1007/s10143-024-02344-w

**Published:** 2024-03-19

**Authors:** Metin Orakdogen, Orkhan Mammadkhanli, Osman Simsek

**Affiliations:** Department of Neurosurgery, Trakya University School of Medicine, Trakya University Hospital Trakya University Hospital, 22030 Balkan Campus, Edirne, Turkey

**Keywords:** ACoA aneurysm, Surgical scoring system, 3D CTA, Aneurysm clipping

## Abstract

**Background:**

The objective was to provide comprehensive preoperative information on both the aneurysm orientation and the side and method of surgical approach for optimal preoperative information and safe clipping using 3D imaging modalities. This was achieved by making an objective risk assessment on the surgical side/method and evaluating its effectiveness with internal validation.

**Materials and methods:**

Radiologic data of 61 ACoA aneurysm patients between 2012 and 2020 were retrospectively analyzed. A scoring system based on five criteria; ACoA aneurysm dome orientation, A1 symmetry/control, perforating artery control, A2 trace orientation, and A2 fork symmetry was developed. The system is designed to align with the most common surgical approaches in ACoA aneurysm surgery. The patients were categorized into three groups based on the scoring results to determine the most appropriate surgical method. Group I was recommended, Group II was less recommended, and Group III was least recommended. Internal validation was performed to assess the system’s effectiveness. Outcomes and complication rates were statistically evaluated.

**Results:**

When the scoring system was utilized, the mean score difference between the first group and the other groups was 2.71 and 4.62, respectively. There was a homogeneous distribution among the groups in terms of age, sex, WFNS, and Fisher scores. Complication occurred in three patients in Group I and nine patients each in Group II and Group III. The further the deviation from the first option, the higher the complication rate (*p* = 0.016), and a significant cause–effect relationship was identified (*p* = 0.021). The ROC curve established a cut-off value of 12.5 points for complications and outcomes.

**Conclusion:**

Our study introduces a new scoring system for ACoA aneurysms, enhancing the use of 3D CTA in daily practice and providing internal validation for the proposed approach. By evaluating objective criteria, this scoring system helps predict surgical risks, prevent complications, and supports personalized evaluation and selection of the surgical approach based on objective criteria.

## Introduction

Anterior communicating artery (ACoA) aneurysm projection is one of the important factors that determine the selection of the most appropriate surgical side, the difficulty level of the surgery, the precautions to be taken during surgical clipping, possible complications, and prognosis. Therefore, it is imperative to know the ACoA projection preoperatively [1–16].

Various factors have been taken into consideration with respect to the side of surgical approach to ACoA aneurysms. Aneurysm dome orientation, A1 dominance, A2 fork orientation, associated aneurysms and variations, surgeon’s experience, nondominant hemisphere/right-handed surgeon are among the most important factors [1, 3, 9, 13, 14, 17–22]. Furthermore, there are many surgical approaches to ACoA aneurysms such as transsylvian, subfrontal, lateral supraorbital, anterior interhemispheric, and orbitozygomatic [3, 9, 13, 14, 17–27].

In our study, we developed a comprehensive and reliable surgical scoring system for ACoA aneurysm clipping, considering essential factors including aneurysm dome orientation, A1 control, perforating artery control, A2 trace orientation, and A2 fork symmetry. This system provides neurosurgeons with valuable insights into safe and risky zones within the surgical field, facilitating precise decision-making during the procedure. Through internal validation, we established the reliability and applicability of our system, aiming to improve surgical outcomes for patients with ACoA aneurysms by providing objective preoperative information and guiding safe clipping procedures.

## Materials and methods

### Study population

After the approval of the local Institutional Committee (number TÜTF-GOBAEK 2023/255), we retrospectively analyzed the radiological data from a cohort of 96 patients with ACoA aneurysms with single/multiple aneurysms, with/without rupture, diagnosed with at least one of 3D CTA, MRI/MRA, or DSA at our University hospital between 2012 and 2020. Out of 96 patients, 61 cases underwent surgical intervention, and their data were employed for internal validation.

### Defining the scoring system

The scoring system was based on the dome orientation of the aneurysm and the vascular variations in the anterior communicating complex, which would allow for the safest placement of the clip by reaching the neck of the aneurysm.

To determine the most appropriate surgical approach and method and to perform risk assessment, a scoring system based on five criteria was established. These criteria include aneurysm dome orientation, A1 control, perforating artery control, A2 trace orientation, and A2 fork symmetry .

A. ***A1 control*** was based on A1 symmetry/control and direct vision without trying to dissect the opposite A1.
B. ***Perforating artery control*** was evaluated as direct view and control, indirect view/control, and no view/no control. Direct vision and control imply direct control without aneurysm neck dissection. Indirect vision and control refer to control with aneurysm neck dissection, while no vision and control indicate no perforating control even with aneurysm dissection.
C. ***Proximal A2/neck control*** was used to demonstrate the relationship between proximal A2s and aneurysm neck according to A2 trace orientation. It was evaluated as bilateral proximal A2/neck or unilateral proximal A2/neck control according to horizontal, vertical, and oblique orientations. In the horizontal orientation, the ACoA complex is positioned without lateral rotation, and both ACoA and its A2 segments are positioned in a coronal plane. In the vertical orientation, extreme rotation of the complex positions the ACoA and its A2 segments along a sagittal plane. An oblique orientation is considered if the position is between horizontal and vertical orientation.
D. ***Aneurysm dome projection*** was classified as contralateral, midline, or ipsilateral based on approach side and technique.
E. **The*A2 fork symmetry*** criterion was based on the relationship between the ipsilateral A2 and aneurysm neck, and was classified as open, symmetrical, and closed.

The “open A2 fork” is characterized by the ipsilateral A2 being positioned more posteriorly than the contralateral A2 on the approach side. Vice versa in the case of the “closed A2 fork”.

**Fig. 1:**
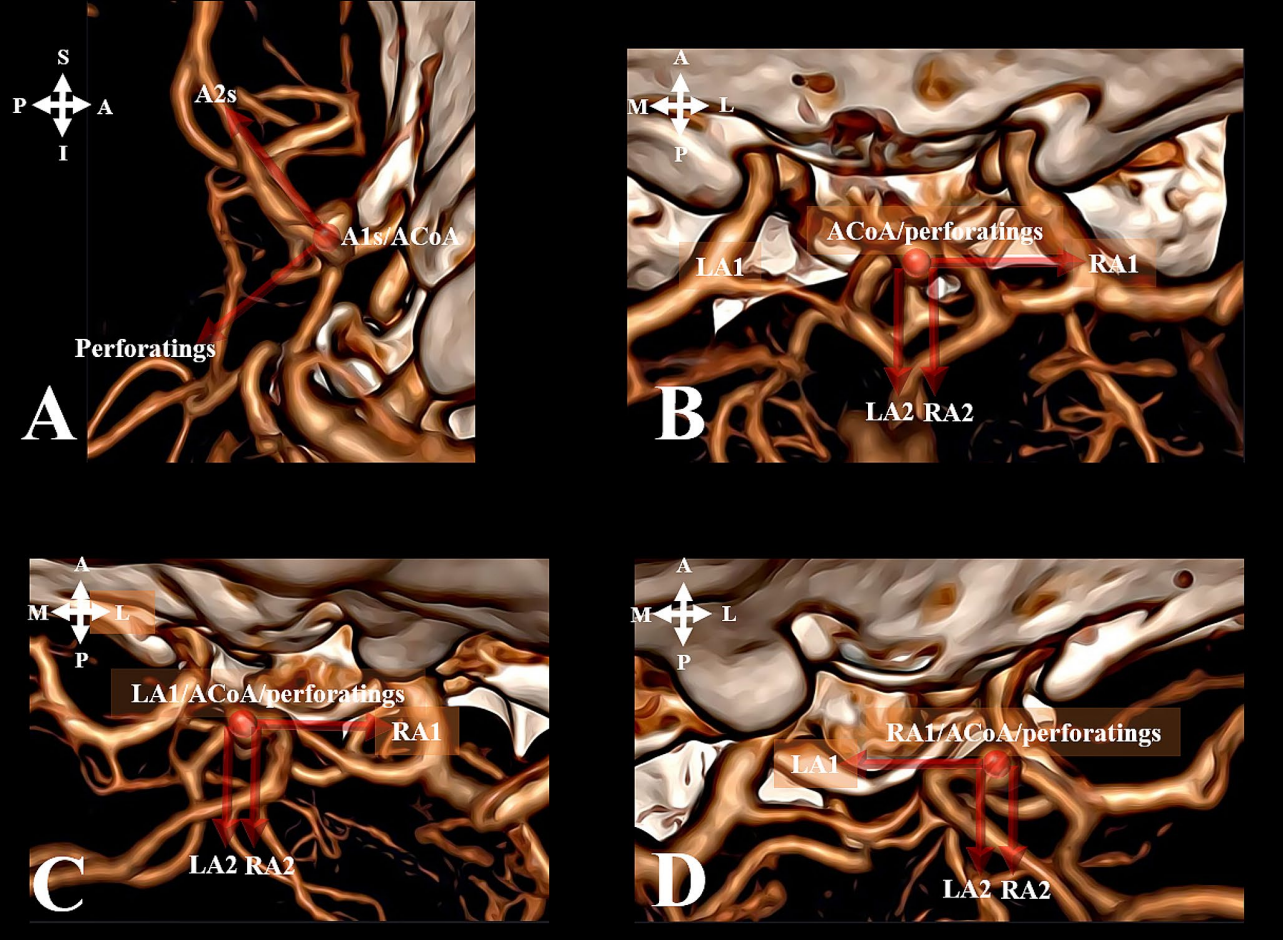
Illustration of the ACoA complex using schematics and angiography based on surgical approach. (S-superior, I - inferior, A - anterior, P - posterior, M- medial, L- lateral) **A**. ACoA complex: vertex down pterional approach **B**. ACoA complex: vertex down basal anterior interhemispheric/subfrontal approach **C**. ACoA complex: vertex down right lateral supraorbital approach **D**. ACoA complex: vertex down left lateral supraorbital approach

**Fig. 2:**
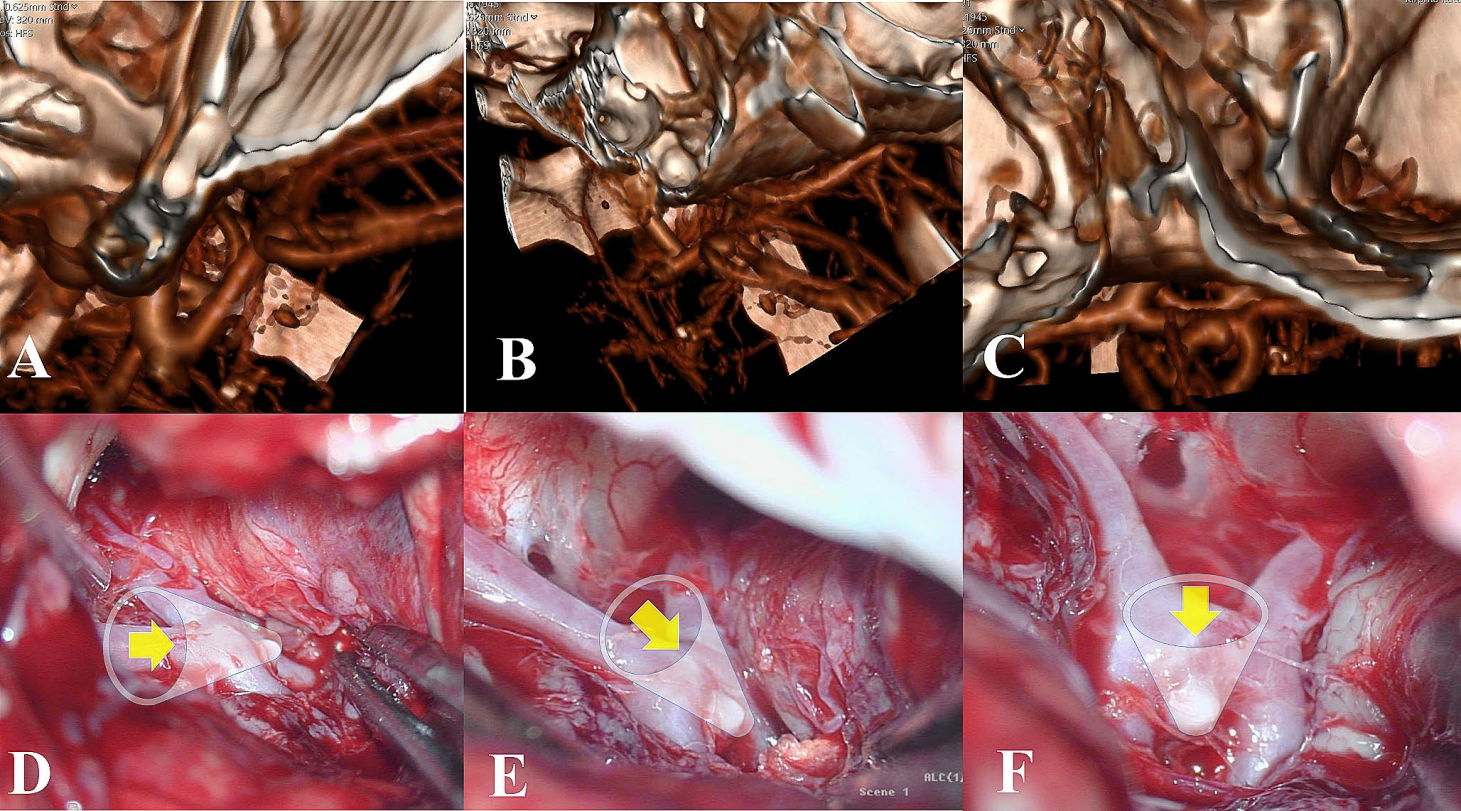
3D CTA (A-C) and intraoperative views (D-F) of superior projection ACoA aneurysm in left pterional, left supraorbital and basal anterior interhemispheric/subfrontal approaches **A**. Left pterional view **B**. Left supraorbital view **C**. Basal anterior interhemispheric/subfrontal view **D**. Intraoperative view of the left pterional approach **E**. Intraoperative view of left supraorbital approach **F**. Intraoperative view of basal anterior interhemispheric/subfrontal approach

As the most common surgical approaches in ACoA aneurysm surgery are pterional, lateral supraorbital, and basal anterior interhemispheric/subfrontal approaches, the scoring system was based on these (right and left). However, all possible surgical sides and methods were assessed on 3D cerebral angiography (Figs. 1 and 2).

**Fig. 3:**
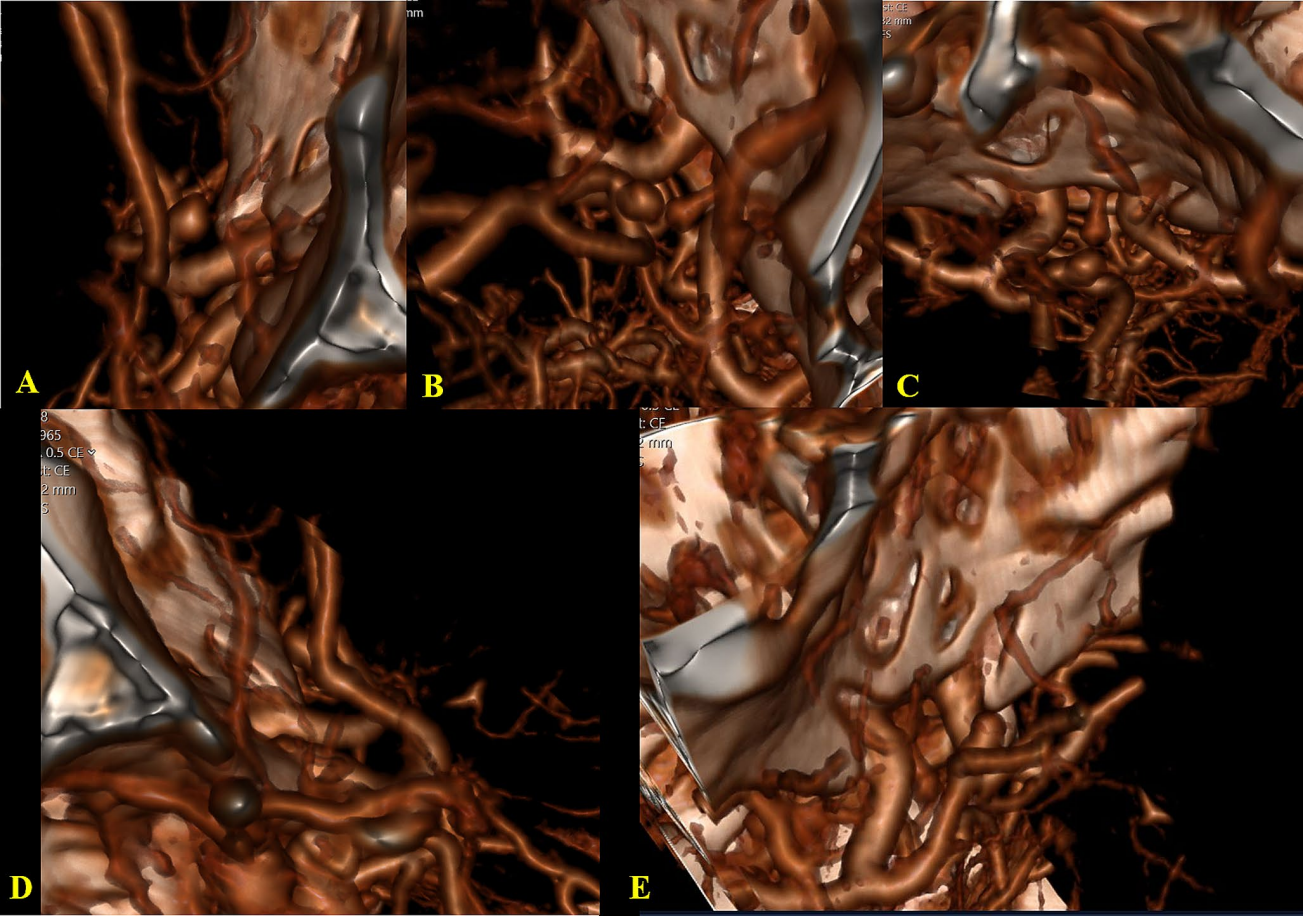
Case example of surgical scale calculation ACoA aneurysm with anterosuperior medial projection. Right A1 hypoplasia and left A2 closed fork present. Surgical approach scores: RPT = 9, RSO = 9, **INT/SF = 13**, LPT = 11, LSO = 10. **A**. Right pterional view (RPT) **B**. Right supraorbital view (RSO) **C**. Basal anterior interhemispheric/subfrontal view (INT/SF) **D**. Left pterional view (LPT) **E**. Left supraorbital view (LSO)

Scoring was performed by considering alternatives for each criterion. The scoring system ranged from 5 to 16 points. The scoring system also considered the surgeon’s use of the dominant hand, the distance of the aneurysm from the skull base, and the complexity of the aneurysm (Table 1; Fig. 3).

**Table 1:** Surgical approach scoring system for ACoA aneurysms

Criteria	Score
**A. A1 control**	
Bilaterally A1	4
Dominant A1	3
Symmetrical A1	2
Nondominant A1	1
**B. Perforating artery control**	
Direct view and control	3
Indirect view/control	2
No view/No control	1
**C. Proximal A2/neck control**	
Bilaterally (horizontal or vertical)	3
Bilaterally (oblique)	2
Unilateral (horizontal or vertical)	1
**D. The aneurysm dome orientation**	
Contralateral	3
Midline	2
Ipsilateral	1
**E. A2 fork symmetry**	
Open A2 fork	3
Symmetrical A2 fork	2
Closed A2 fork	1

Note:

For right-handed surgeons, 1 point is added to the total score for right-sided approaches and 1 point is subtracted for left-sided approaches. The opposite is done for left-handed surgeons

In patients with a skull base–aneurysm distance > 10 mm, one point is added to the total score in the basal anterior interhemispheric/subfrontal approach and one point is subtracted in other approaches

For aneurysm diameter > 10 mm and complex aneurysms, 1 point is subtracted from the total score in all approaches

### Internal validation group

Out of 61 patients, 56 cases were ruptured, and 5 unruptured. These patients were assessed as ruptured and all patients (ruptured and unruptured). The patients were categorized into three groups based on the surgical approach used;

**Group 1** consists of cases where the intervention suggested by the surgical scoring system is the first option (highest score).

**Group 2** consists of cases where the intervention suggested by the surgical scoring system is the second option (second highest score).

**Group 3** consists of cases where the intervention suggested by the surgical scoring system is the other options (lowest score).

The surgeries were performed by more than one experienced surgeon. Each group may have multiple surgical approach options, including pterional, basal anterior interhemispheric/subfrontal, and lateral supraorbital, depending on the scores given according to our surgical scoring system.

The mean score differences between the groups for the approach recommended by the scoring system and the preferred approaches were analyzed.

Outcomes and complication rates (perforating and parent artery infarction) were investigated in these groups. Glasgow Outcome Scale (GOS) 5 − 4 was considered a favorable outcome and GOS 3 − 1 as an unfavorable outcome.

### Statistical analyses

Statistical analyses were performed using Jamovi (version 2.3). The results were presented as mean ± standard deviation for parametric continuous variables and as percentage for categorical variables. Two-group comparisons were made using student’s t-test for parametric variables and χ² test and Fisher’s exact test for categorical variables. One-way analysis of variance and Kruskal–Wallis tests were used to compare the means or medians of a continuous variable (age) between different groups. Mantel–Haenszel trend test was used to determine whether there was a linear trend between two categorical variables. A *p*-value of ≤ 0.05 was considered significant. In addition, a receiver operating characteristic (ROC) curve was generated to evaluate the prediction of postoperative complications and GOS by the surgical scale.

## Results

**Fig. 4:**
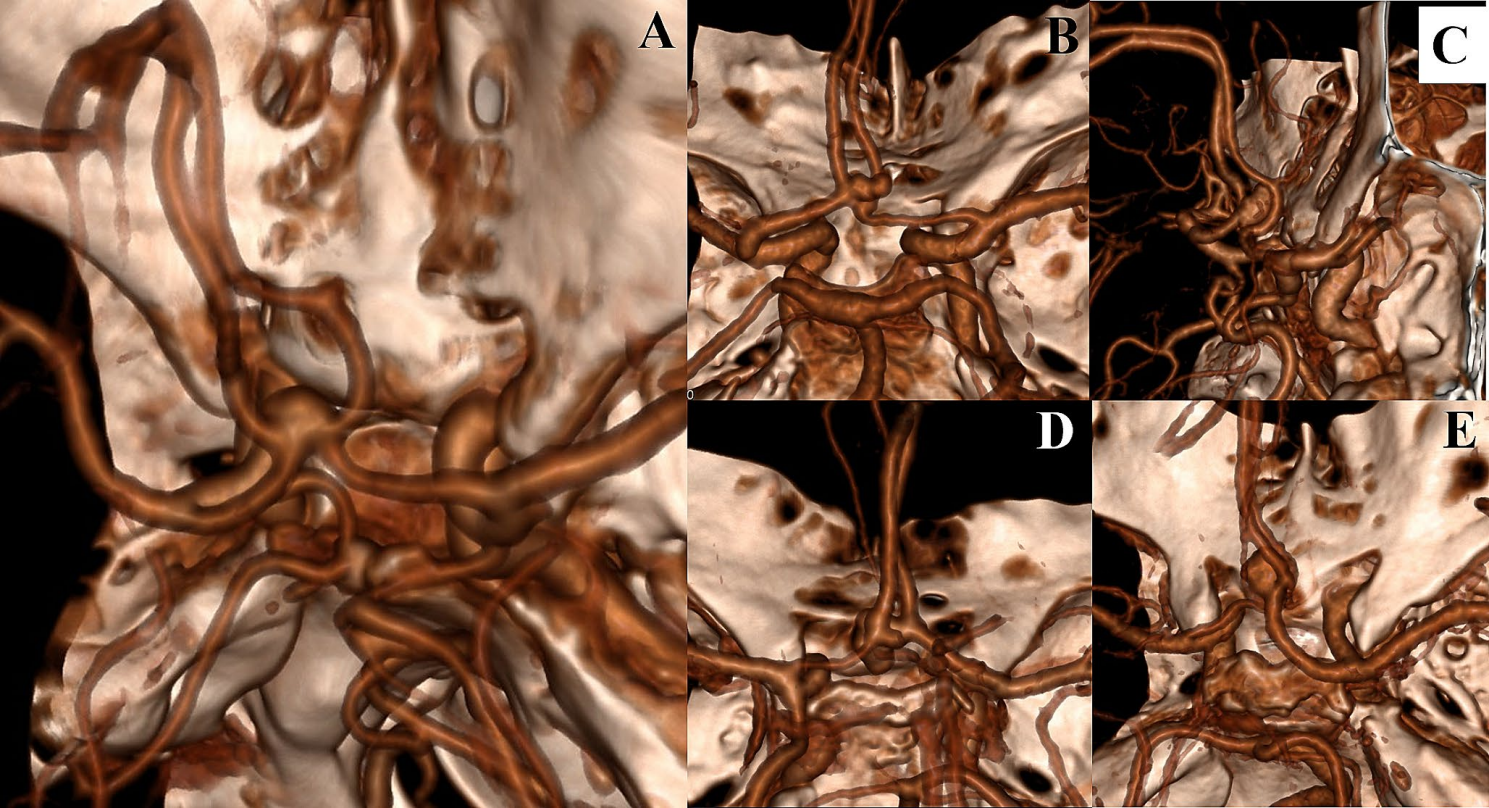
**Types of A1 and A2 Variations** **A**. ACoA aneurysm: Symmetric A1 **B**. ACoA aneurysm: Asymmetric A1 **C**. ACoA aneurysm: Closed A2 fork **D**. ACoA aneurysm: A2 oblique orientation and Asymmetric A1 **E**. ACoA aneurysm: A2 vertical orientation and Asymmetric A1

Among the 96 patients included in the study, 76 had only ACoA aneurysms, while 20 had multiple aneurysms. Demographically, there were 39 males and 22 females with a mean age of 54.54 ± 11.8, ranging from 29 to 82 years. Aneurysm diameters ranged between 2 and 13 mm, with an average 6.62 mm ± 2.43. A1 and A2 variations (A1 control/diameter symmetries, aneurysm dome orientations, A2 trace orientations, and A2 fork symmetries) for all cases (96 patients) are given in Table 2 (Fig. 4).

**Table 2:** A1 and A2 variations (A1 diameter asymmetry, A1 symmetry / Aneurysm projection, A2 trace orientation, A2 fork asymmetry)

Anterior cerebral artery A1 and A2 variations			Number of case (%)
A1 symmetry / Aneurysm projection *n* = 96	Asymmetrical *n* = 78 (81,25%)	Medial	47 (60.25)
		Lateral	4 (5.12)
		Midline	24 (30.77)
		Bilobed	3 (3.84)
	Symmetrical *n* = 18 (18,75%)	Medial	9 (50)
		Lateral	1 (5.55)
		Midline	8 (44.44)
		Bilobed	-
A1 diameter asymmetry	Left A1 *n* = 78	Dominance	41
		Prominent hypoplasia	21
		Aplasia	8
		Only asymmetric	8
	Right A1 *n* = 78	Dominance	37
		Prominent hypoplasia	14
		Aplasia	18
		Only asymmetric	9
A2 variations	A2 trace orientation *n* = 96	Classic	57 (59.37)
		Oblique	24 (25)
		Vertical	15 (15.62)
	A2 fork *n* = 96	Symmetrical	73 (76)
		Asymmetric (closed/open)	23 (24)

A total of 61 patients underwent surgical treatment. Standard pterional approach was used as the surgical method. In the choice of the operation side, the dominant A1 side was preferred. In the presence of concomitant aneurysms, side choice was performed taking these aneurysms into account.

The aneurysm projection was evaluated using a 3D classification system [28]. The approach methods were then analyzed according to these projections (Table 3).

**Table 3:** Relationship between aneurysm projections and first approach option based on surgical scoring system

Projection	Pterional (*n* = 16)	Supraorbital (*n* = 10)	Basal Anterior Interhemispheric//Subfrontal (*n* = 44)
Anterior-median	2	1	3
Anterior-lateral	1	-	-
Anterior-medial	1	-	4
Anterosuperior-medial	5	4	10
Anterosuperior-lateral	-	1	1
Anterosuperior-median	1	-	5
Anteroinferior-median	1	1	2
Anteroinferior	-	-	1
Anteroinferior-medial	1	-	3
Superior-median	-	-	4
Superior-medial	1	-	2
Posterior-medial	1	1	4
Posterosuperior-medial	1	1	-
Posterosuperior-median	1	-	-
Posteroinferior-medial	-	-	1
Complex	-	1	4

Based on the surgical scoring, 44 patients were suitable for the basal anterior interhemispheric/subfrontal approach (INT/SF), while 16 patients were suitable for the pterional approach (10 on the right, 6 on the left), and 10 patients for the right supraorbital (RSO) approach as the first approach option (4 patients had 2 options and 3 patients had 3 options with the maximum score). The surgical approach used was the first option in 15 (24.6%) patients, the second option in 33 (54.1%) patients, and the third option in 13 (21.31%) patients. The mean score difference in the surgical approach was 2.71 in Group II and 4.62 in Group III compared to Group I.

In the 61 operated cases, the rate of favorable outcome was 13/15 (86.7%) in Group I, 24/33 (72.7%) in Group II, and 10/13 (76.9%) in Group III.

### Complications

In Group 1, there were two caudate and one caudate + hypothalamic infarct (three patients), in Group 2, there were five caudate, two hypothalamic, one hypothalamic + caudate infarct, and one hypothalamic + caudate + parent artery (distal anterior cerebral artery (DACA)) infarct in a patient with prominent vasospasm (nine patients), and in Group 3, there were six caudate, one DACA, one bilateral hypothalamic + caudate infarct, and one hypothalamic + DACA infarct (nine patients). Totally, there were 21 patients with perforating and parent artery infarcts.

Information about the cases in the study context is summarized in Table 4.

**Table 4:** Summary of patients included in the study

Case	Age and gender	RPT	LPT	RSO	LSO	INT/SF	Prefered choice	Choice	Comp.	Outcome
1	45, M	14	8	12	4	9	RPT 14	1	No	Favorable
2	39, M	14	10	11	9	13	RPT 14	1	No	Favorable
3	60, M	10	13	10	12	12	LPT 13	1	No	Favorable
4	49, M	12	8	12	7	12	**RPT 12**	**1**	**Yes**	Favorable
5	64, M	14	6	12	7	11	**RPT 14**	**1**	**Yes**	Favorable
6	58, M	8	11	7	10	10	LPT 11	1	No	Favorable
7	61, F	15	8	13	9	14	RPT 15	1	No	**Unfavorable**
8	58, F	14	10	14	10	14	RPT 14	1	No	Favorable
9	38, M	15	9	14	7	15	RPT 15	1	No	Favorable
10	38, M	14	6	11	7	12	RPT 14	1	No	Favorable
11	57, M	14	5	12	7	14	RPT 14	1	No	**Unfavorable**
12	52, M	9	12	11	9	11	**LPT 12**	**1**	**Yes**	Favorable
13	70, M	11	13	10	9	12	LPT 13	1	No	Favorable
14	66, M	5	15	6	14	15	LPT 15	1	No	Favorable
15	59, M	13	9	13	8	12	RPT 13	1	No	Favorable
16	34, M	13	7	15	7	11	RPT 13	2	No	Favorable
17	64, F	9	9	9	9	11	LPT 9	2	No	Favorable
18	44, F	11	9	11	9	13	RPT 11	2	No	**Unfavorable**
19	58, M	6	8	6	8	12	**LPT 8**	**2**	**Yes**	Favorable
20	63, M	12	8	10	7	14	RPT 12	2	No	Favorable
21	65, M	9	10	8	7	13	LPT 10	2	No	Favorable
22	44, F	7	11	8	9	12	**LPT 11**	**2**	**Yes**	**Unfavorable**
23	67, M	9	9	8	8	14	**LPT 9**	**2**	**Yes**	Favorable
24	37, M	11	8	10	7	14	RPT 11	2	No	Favorable
25	50, F	10	12	9	12	12	**RPT 10**	**2**	**Yes**	Favorable
26	70, F	11	8	12	7	10	**RPT 11**	**2**	**Yes**	Favorable
27	46, F	12	7	14	8	14	**RPT 12**	**2**	**Yes**	Favorable
28	55, F	7	10	6	10	11	**LPT 10**	**2**	**Yes**	**Unfavorable**
29	67, M	8	10	7	9	12	**LPT 10**	**2**	**Yes**	Favorable
30	59, M	11	8	10	7	14	RPT 11	2	No	Favorable
31	65, M	11	13	11	11	15	LPT 13	2	No	Favorable
32	78, F	11	9	11	9	14	RPT 11	2	No	**Unfavorable**
33	52, M	8	8	8	8	13	RPT 8	2	No	Favorable
34	64, M	10	9	12	4	10	RPT 10	2	No	Favorable
35	55, M	11	8	14	7	11	RPT 11	2	No	Favorable
36	64, F	11	9	9	11	13	RPT 11	2	No	Favorable
37	67, F	12	9	11	9	14	RPT 12	2	No	**Unfavorable**
38	82, F	9	9	8	8	13	RPT 9	2	No	**Unfavorable**
39	32, M	12	8	11	7	15	RPT 12	2	No	Favorable
40	58, F	13	9	13	7	15	RPT 13	2	No	**Unfavorable**
41	61, M	9	10	10	10	15	LPT 10	2	No	Favorable
42	60, M.	9	11	8	10	13	LPT 11	2	No	Favorable
43	57, F.	11	11	10	9	14	**RPT 11**	**2**	**Yes**	**Unfavorable**
44	34, F.	12	9	10	10	14	RPT 12	2	No	**Unfavorable**
45	41, M.	10	11	10	8	15	LPT 11	2	No	Favorable
46	29, M.	7	10	9	10	15	LPT 10	2	No	Favorable
47	42, M.	13	6	13	7	14	RPT 13	2	No	Favorable
48	46, M.	9	10	8	10	14	LPT 10	2	No	Favorable
49	44, M.	13	9	9	9	14	**LPT 9**	**3**	**Yes**	Favorable
50	66, F.	10	8	13	7	12	RPT 10	3	No	Favorable
51	46, M.	11	9	12	7	14	**RPT 11**	**3**	**Yes**	Favorable
52	59, M.	9	10	8	9	13	**RPT 9**	**3**	**Yes**	Favorable
53	49, F.	9	8	9	7	14	**LPT 8**	**3**	**Yes**	Favorable
54	74, F.	10	12	9	9	14	**RPT 10**	**3**	**Yes**	Favorable
55	52, F.	10	13	8	12	14	**RPT 10**	**3**	**Yes**	**Unfavorable**
56	47, M.	10	10	10	11	15	RPT 10	3	**Yes**	Favorable
57	44, F.	10	7	14	7	11	**RPT 10**	**3**	**Yes**	**Unfavorable**
58	68, M.	9	10	11	12	15	RPT 9	3	No	**Unfavorable**
59	52, M.	7	11	7	9	15	LSO 9	3	No	Favorable
60	47, M.	11	8	10	10	10	**LPT 8**	**3**	**Yes**	Favorable
61	55, M.	5	7	10	10	16	RPT 5	3	No	Favorable

**Abbreviations**: RPT - right pterional approach; LPT - left pterional approach; RSO - right supraorbital approach; LSO - left supraorbital approach; INT/SF - basal anterior interhemispheric/subfrontal. Comp. - complication related to perforating and parent artery infarct; Outcome - Favorable (GODS 4–5), unfavorable (GOS 1–3)

### Results of statistical analysis

Statistical analyses were performed in terms of age, sex, World Federation of Neurological Surgeons (WFNS) grading, Fisher’s score, complication rate and favorable GOS, and recommended surgical options (ruptured and all (ruptured and unruptured) surgically treated group) (Table 5).

**Table 5:** Statistical analysis of ruptured and total (ruptured and unruptured) aneurysms

			Surgical options					*P* value	
		1st Choice		2nd Choice		3rd Choice			
		*n* = 12	*n* = 15	*n* = 31	*n* = 33	*n* = 13	*n* = 13	Ruptured	Total
		1 2	1 2	1 3	1 3	2 3	2 3	*n* = 56	*n* = 61
Age (*p* value)		0.482	0,968	0.933	0,959	0.858	0,968	0.5*	0.946*
WFNS (*p* value)		0.592	0.999	0.803	0.311	0.328	0.213	0.301*	0.216*
Fisher (*p* value)		0.600	1.000	0.757	0.271	0.145	0.089	0.155 *	0.112*
		**Ruptured**	**Total**	**Ruptured**	**Total**	**Ruptured**	**Total**		
Gender	M	10 (83,3%)	13 (86.7%)	17 (54.8%)	19 (57,6%)	8 (61,5%)	8 (61,5%)	0,223	0,132
	F	2 (16.7%)	2 (13.3%)	14 (45.2%)	14 (42,4%)	5 (38,5%)	5 (38,5%)		
Favorable Outcome	1	10 (83.3%)	13 (86.7%)	22 (71%)	24 (72,7%)	10 (76.9%)	10 (76.9%)	0,843	0,657
	0	2 (16.7%)	2 (13.3%)	9 (29%)	9 (27.3%)	3 (23.1%)	3 (23.1%)		
Complication	1	3 (25%)	3 (20%)	9 (29%)	9 (27.3%)	9 (69.2%)	9 (69.2%)	**0,033**	**0,016**
	0	9 (75%)	12 (80%)	22 (71%)	24 (72.7%)	4 (30.8%)	4 (30.8%)		

*n* = number of patients, m – male, f – female, Favorable Outcome 1 (GOS 4–5), 0 - (GOS 1-2-3), Complication 1 (if present), 0 (absent)

There was no statistically significant difference between the surgical option groups in terms of age (*p* = 0.5 and 0.946), sex (*p* = 0.223 and 0.132), WFNS grading (*p* = 0.301 and 0.216), and Fisher’s score (*p* = 0.155 and 0.112). The distribution of these factors was homogeneous among the groups.

Although there was no statistically significant relationship between favorable outcome and surgical option (*p* = 0.843 and 0.657), the percentage of favorable outcome was higher in Group I. There was a statistically significant correlation between surgical options and complication rates (*p* = 0.033 and 0.016). This association between the two variables was seen with a high degree of confidence (ruptured: χ² = 5.31, *p* = 0.021; total group: χ² = 6.98, *p* = 0.008).

**Fig. 5:**
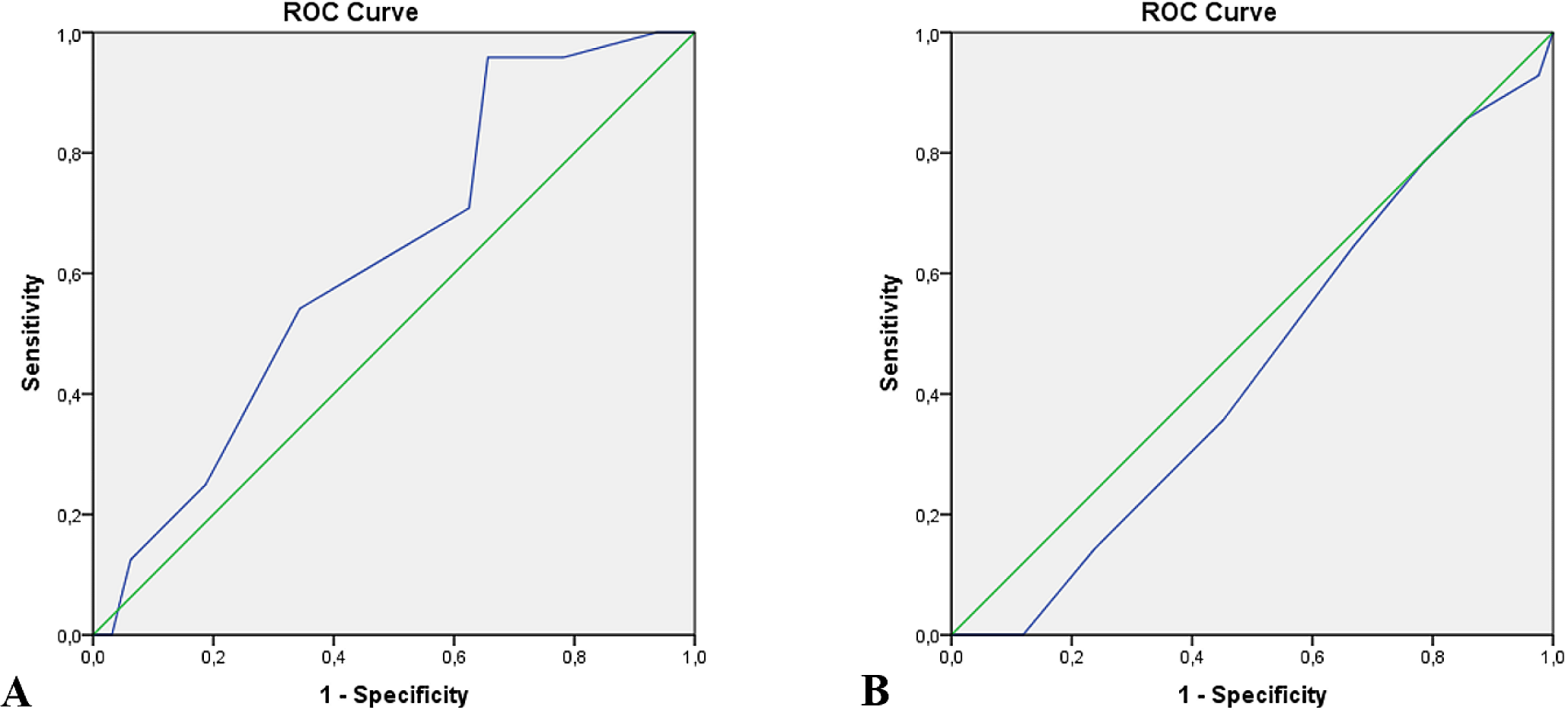
Receiver operating characteristic (ROC) curves of surgical scoring system for complications (**A**) and outcome (**B**)

ROC analysis was performed on the inverted score (15-score) of the selected approach to predict complications and unfavorable outcomes. The cut-off point for complications was determined to be 12.5, with a sensitivity of 95.8% and a specificity of 34.4%. The cut-off point for unfavorable outcomes was also found to be 12.5, with a sensitivity of 78.6% and a specificity of 21.4% (Fig. 5).

## Discussion

There are challenges in ACoA aneurysm surgery owing to different factors. In our study, by considering A1 control, A2 trace orientation, A2 fork symmetry, aneurysm dome orientation, and perforating artery control, a scoring system was created to provide maximum preoperative information for safe clipping to enable a more objective risk assessment of the side/method of approach. The internal validation revealed that fewer complications were met when the approach option suggested by this system was used.

### Advantages of 3D CTA and DSA

Advances in 3D technology offer enhanced accuracy in evaluating aneurysm morphology and projections. Unlike conventional DSA, 3D CTA and DSA provide a 360-degree global, coronal, sagittal, and axial view, enabling detailed assessments of ACoA aneurysm relationships with proximal A1 and A2 outlets. Variations in A2 tracing and fork symmetry are better visualized. The greatest advantage is the ability to preoperatively decide the most appropriate surgical approach by visualizing aneurysms and vascular structures from the desired surgical head position.

### Variations of the ACoA complex

Variations of the ACoA complex are frequent and may affect the side and method of surgical approach. These include A1 asymmetry, A2 trace orientation, and A2 fork symmetry [3, 9, 11, 14, 17–20, 29, 30]. Therefore, each aneurysm should be evaluated individually. A1 asymmetry was found in 81.25% of our patients .

In the classical ACoA complex, the A2 segments and ACoA do not show lateral rotation, and they are situated in the coronal plane. However, in cases where there is excessive rotation of the complex, the ACoA and A2 segments are positioned in the sagittal plane [11].

**Fig. 6:**
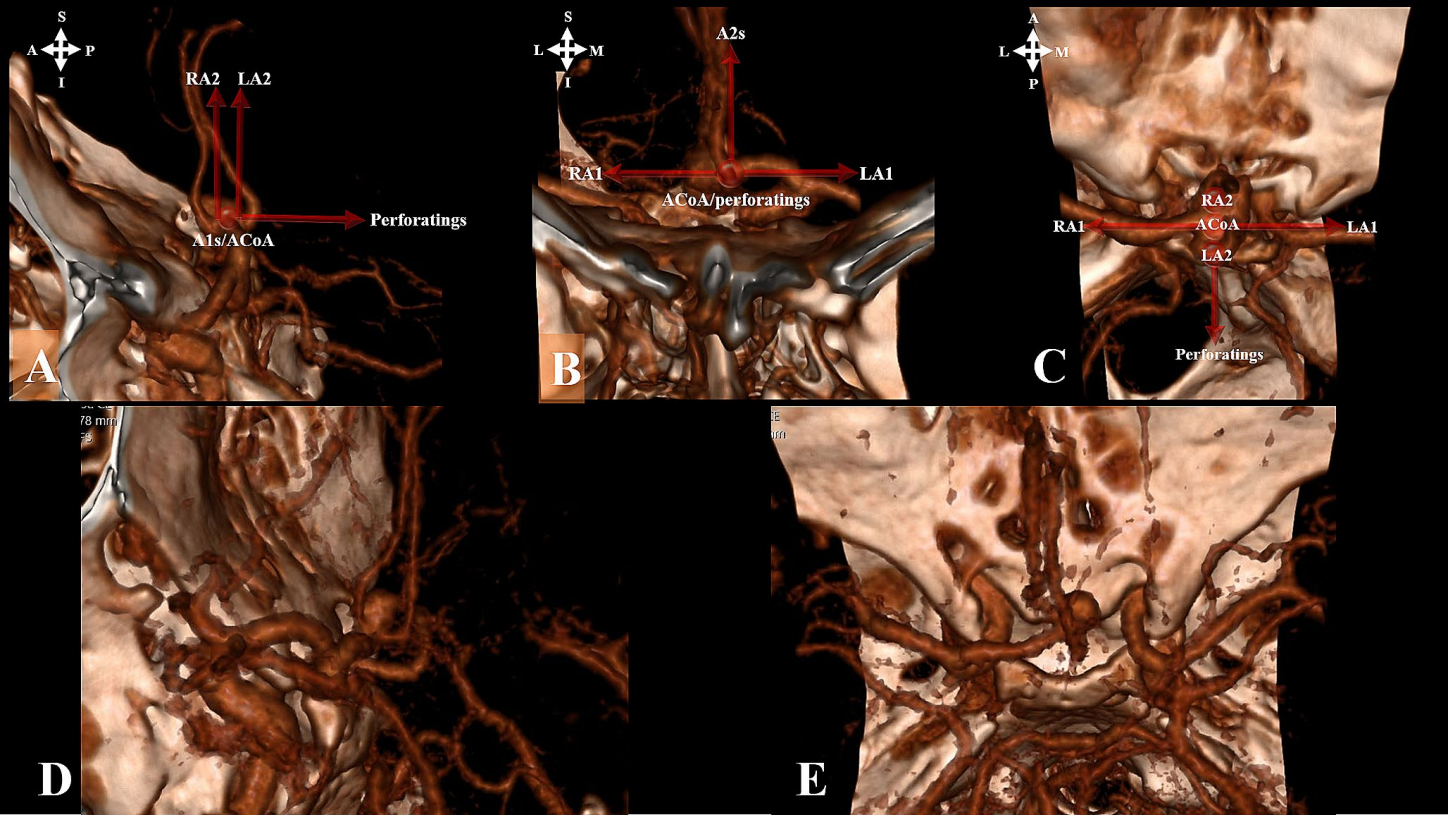
**Schematic and Angiographic Views of the ACoA Complex in Sagittal, Coronal and Axial Planes with Vertical A2 Orientation (A-C). CT angiography views of pterional and basal anterior interhemispheric/subfrontal approaches in the presence of vertical A2 orientation in superior projection ACoA aneurysm (D,E)**. **(S-superior, I - inferior, A - anterior, P - posterior, M- medial, L- lateral)** **A**. Vertical A2 orientation in the sagittal plane **B**. Vertical A2 orientation in the coronal plane **C**. Vertical A2 orientation in the axial plane **D**. Left pterional approach **E**. Basal anterior interhemispheric/subfrontal approach

In the presence of vertical A2 trace orientation, aneurysm projections are also displaced owing to the 90-degree shift of the classical orientation. The anterior–posterior projection is replaced by the medial–lateral and the medial–lateral projection by the anterior–posterior. In superior projection aneurysms, the classical A2 orientation is a disadvantage for aneurysm visualization and clipping with the pterional approach and an advantage for the basal interhemispheric/subfrontal approach, while the opposite is true for the vertical A2 orientation (Fig. 6**).** In our study, classical A2 tracing was detected in 59.37% of the patients. Vertical A2 trace orientation was seen in 15.62% and oblique A2 trace orientation in 25% of the cases.

The orientation of the A2 fork refers to the relationship between the plane of the A2 fork and the midsagittal plane, where one A2 segment is positioned in front and the other behind. The “closed A2 plane” is described as the situation where the ipsilateral A2 is located more anteriorly, causing the A1-A2 junction and the A2 to obscure the neck of the aneurysm [17, 18, 29]. Asymmetric (open/closed) A2 fork was found in 24% of our patients with or without superior projection aneurysm.

### Choice of surgical side and approach in ACoA aneurysms

The surgical approach to ACoA aneurysms depends on many factors, such as presence of rupture, aneurysm size, dome orientation, A1 length, tracing and dominance, A2 fork orientation, height of the ACoA complex from the base of the skull, neurovascular variations, presence of concomitant aneurysm, presence and extension of atherosclerosis in the parent artery and aneurysm base, presence of intracerebral/intraventricular hemorrhage, existing neurological deficits, previous surgeries, experience of the surgeon, and nondominant hemisphere/right-handed surgeon [19].

The surgical approach methods include transsylvian, subfrontal, lateral supraorbital, unilateral/bilateral/basal anterior interhemispheric, cranioorbitozygomatic, transorbital, skull base approaches, and their keyhole modifications [3, 19, 20, 27]. The view of the ACoA complex and its aneurysm varies with the surgical approach [27]. 

Yasargil et al. [22] used the standard pterional craniotomy and recommended the right side for right-handed surgeons, with exceptions. According to Solomon et al. [25], standard pterional craniotomy is sufficient.

According to Hernesniemi et al. [19], the most important factor in the selection of surgical side is A1 dominance, hence the lateral supraorbital approach and dominant A1 side are used. The anterior interhemispheric approach is preferred in aneurysms with anterior/posterior projection and in the presence of high ACoA complex.

In the pterional approach, it has been reported that A1 dominance is more important in inferior projection aneurysms and the dominant A1 side should be used [1, 20]. In the posterior projection, a closed A2 plan should be preferred [1]. However, other authors have stated that the dominant A1 side is a better choice for approaching ACoA aneurysms, whether the A2 plan is open or closed [17].

Nondominant side or A1 dominance can be used for side choice. Particular attention should be paid to the superior projection and the vertical A2 trace [3]. Other authors have employed the supraorbital keyhole approach [17, 26]. In the inferior projection, the lateral approach is more appropriate than the supraorbital approach [24].

A2 fork symmetry is important in determining the surgical approach and complications [14, 18, 20, 29]. When dealing with aneurysms projecting towards the rear, Chen et al. [18] recommend selecting the side where the A2 segment is positioned in front, considering potential obstruction by the ACoA complex. In such cases, the A2 segment on the same side should be carefully dissected from a posterior approach, possibly involving the removal of the gyrus rectus to achieve extensive exposure of the aneurysm base.

The importance of A2 plan in superior projection has been emphasized in terms of necessity of gyrus rectus resection, aneurysm neck residual, and postop complications [14, 20]. In the presence of a closed A2 plan in dorsal projection and high positioned aneurysms, the use of skull base techniques or interhemispheric approach instead of normal pterional craniotomy will improve postop patient outcome [14]. Liu et al. [21] suggested that the pterional approach from the opposite side of A1 dominance can provide good proximal control and is more convenient, safe, and effective.

Sekhar et al. [13] preferred the dominant A1 side and the nondominant hemisphere side in the presence of symmetric A1 and routinely used the frontoorbital pterional approach and the combined subfrontal and interhemispheric approach in giant aneurysms. In accordance with the ‘A2 fork,’ Chen L et al. [18] routinely selected the non-dominant hemisphere’s side as the surgical approach if the A2 fork had a non-dominant direction, and both A1 segments were of equal diameter. Riina et al. [24] prefer the orbitozygomatic or modified orbitozygomatic approach, and Petraglia et al. [23] use the modified subfrontal approach with unilateral frontal craniotomy.

Starting from the mid-1970s, many institutions have adopted the interhemispheric approach for treating ACoA aneurysms, particularly when these aneurysms are situated deep within the interhemispheric fissure, especially if they extend in a superior direction. Furthermore, the interhemispheric approach offers advantages in terms of avoiding rectus gyrus resection, it provides adequate orientation of the ACoA complex structures and exposure of the aneurysm with less dissection, particularly in ACoA aneurysms with a skull base distance greater than 10 mm. According to our scoring system, 44 patients were found to be suitable for the basal anterior interhemispheric/subfrontal approach. While the pterional approach is the most utilized method in ACoA aneurysm surgery, neurosurgeons should not restrict themselves to it alone. They should have knowledge and awareness of the surgical risks associated with this approach and also be informed about alternative methods, such as the basal interhemispheric/subfrontal, lateral supraorbital, and even orbitozygomatic approaches, which can serve as alternatives.

In our study, in accordance with the above information, the basal anterior interhemispheric/subfrontal approach was predominantly recommended for aneurysms including anterior and superior projections. This supports the usefulness of our scoring system for projection-based surgical approach.

In our opinion, it is incorrect to generalize that all ACoA aneurysms should operate by the nondominant hemisphere or A1 dominance side or that only pterional, subfrontal, or orbitozygomatic approach may be sufficient. Choice of the surgical method based solely on aneurysm projection should consider the presence of a standard ACoA complex without variation. For example, if an aneurysm has a superior/posterior projection with vertical A2 orientation, the basal anterior interhemispheric/subfrontal approach may pose surgical difficulties. The diversity and frequency of ACoA complex variations, and the possibility of multiple coexisting variations, imply that relying on a single criterion while ignoring others may lead to surgical challenges and complications. Establishing a scoring system that considers the pros and cons of each potential surgical side and approach, evaluating objective criteria on a case-by-case basis, is essential. CTA offers advantages over 3D DSA and 3D MRA, providing clear visualization of the bone structure and ACoA complex, aligning with the planned surgical approach and desired angle.

In our study the predominant surgical approach applied in our patients was option two (54.1%). An average difference of 2.7 points was found between surgical approaches using the first and second options. In the unruptured group (five patients), first and second options were applied. All patients in this group had a good outcome and no perforating artery infarction was detected. In patients with ruptured aneurysms, perforating and parent artery infarction was found to be more common when moved away from the first option (*p* = 0.016) and the causal relationship (Mantel–Haenszel test) was also significant (*p* = 0.021). Moreover, we found that although the specificity of ROC analysis was low, the cutoff value for both complications and GOS was 12.5. These data suggest that the probability of complications and unfavorable outcomes may increase in cases with a score of 12 and below. Although there was no statistically significant difference, the favorable outcome was better among unruptured patients and those in Group I. Considering that caudate infarcts are mostly associated with vasospasm and hypothalamic infarcts with the surgical clipping method, the hypothalamic infarction, which was more common in Groups II and III, may be directly related to the surgical choice. Although the number of patients is small, complication rate and poorer outcome in patients with ruptured aneurysms compared with unruptured cases may be explained by the high surgical risk and complications related to vasospasm.

The experience of the surgeon is undoubtedly important in the selection of the side and the surgical method in ACoA aneurysm surgery. However, it is advisable for the surgeon not to limit themselves to a single approach method. Instead, it is recommended that the surgeon evaluate each case individually and use the method that will minimize the risk of complications. This is particularly crucial for less experienced surgeons.

Currently, there is a growing trend towards endovascular treatment for ACoA aneurysms due to advancements in endovascular technologies. However, surgical treatment remains crucial, particularly in underdeveloped and developing countries where access to endovascular treatment is limited and inadequate. Furthermore, it is imperative that every neurosurgeon is knowledgeable about surgical treatment methods and can apply them, when necessary, particularly in cases where endovascular treatment is not feasible, or complications may develop from endovascular treatment.

### Limitations of the study

The limitations of our study include the fact that it was retrospective and consisted mostly of patients with ruptured aneurysms. There was more than one surgeon, and the number of patients was relatively small. It would be appropriate to evaluate the clinical implications of the study with a large case series to be created using the surgical approach scoring system proposed in this study.

## Conclusion

The proposed surgical approach scoring system provides generalization of surgical approaches based on projection and may allow case-by-case evaluation by considering objective criteria that influence the choice of approach side and method. This allows for the prediction of potential surgical risks and complications associated with the surgical side and approach.

In addition, the following points are to be noted:

- The total score may indicate not only the approach with the least risk but also the level of difficulty if surgery other than the recommended approach is chosen,
- With the scoring system, it is possible to predict preoperatively which approach will be difficult for which criterion and the degree of this difficulty (the risks to be taken based on these criteria should be considered in the surgical approach to be applied),
- Combined approaches should also be considered in aneurysms whose score indicates high risk for all approaches, especially complex aneurysms.

In summary, our study has shed light on the complex nature of surgical approaches to ACoA aneurysms and has proposed a comprehensive and reliable scoring system to aid in surgical decision-making. Through the use of 3D technology, we have developed a new preoperative surgical scoring system that enhances the understanding of critical factors affecting the surgical approach.

## Data Availability

The data and materials supporting the findings of this study are available from *Trakya University PACS system* upon reasonable request. Researchers interested in accessing the data and materials used in this study should contact morakdogen@gmail.com for further information.
